# Reply to Fattorini and Borges: Conditional CIs and sensitivity analysis characterize uncertainty in global insect richness estimates

**DOI:** 10.1073/pnas.2631365123

**Published:** 2026-10-05

**Authors:** Robert K. Colwell, Laura Melissa Guzman, Dirk Steinke, Anne Chao, Daniel H. Janzen, Winnie Hallwachs, Austin Baker, José L. Fernández-Triana, Paul D. N. Hebert, Frank Joyce, Robert Puschendorf, Donald L. J. Quicke, Rodolphe Rougerie, M. Alex Smith, Nelson Zamora, Michael J. Sharkey

**Affiliations:** ^a^ Department of Ecology and Evolutionary Biology, University of Connecticut, Storrs, CT 06269-3043; ^b^Entomology Section, University of Colorado Museum of Natural History, Boulder, CO 80309-0001; ^c^ Department of Entomology, Cornell University, Ithaca, NY 14853-2601; ^d^ Centre for Biodiversity Genomics, Department of Integrative Biology, University of Guelph, Guelph, ON N1G2W1, Canada; ^e^ Institute of Statistics, National Tsing Hua University, Hsin-Chu 30043, Taiwan; ^f^ Department of Biology, University of Pennsylvania, Philadelphia, PA 19104-6018; ^g^ Department of Entomology, Natural History Museum of Los Angeles County, Los Angeles, CA 90007; ^h^Canadian National Collection of Insects, Ottawa, ON K1A 0C6, Canada; ^i^Monteverde Institute, Monteverde, Puntarenas 60109, Costa Rica; ^j^ School of Biological and Marine Sciences, University of Plymouth, Plymouth PL4 8AA, United Kingdom; ^k^ Department of Biology, Faculty of Science, Center of Excellence in Integrative Insect Ecology, Chulalongkorn University, Bangkok 10330, Thailand; ^l^Department of Origins and Evolution, Institut de Systématique, Évolution, Biodiversité, Muséum National d’Histoire Naturelle, Centre National de la Recherche Scientifique, École Pratique des Hautes Études—Université Paris Sciences and Lettres, Sorbonne Université, Université des Antilles, Paris F-75005, France; ^m^ Department of Integrative Biology, University of Guelph, Guelph, ON N1G2W1, Canada; ^n^ Escuela de Ingeniería Forestal, Instituto Tecnológico de Costa Rica, Cartago 159-7050, Costa Rica; ^o^The Hymenoptera Institute, Lexington, KY 40502

We welcome the comments of Fattorini and Borges (henceforth F&B) (1) regarding the important issue of uncertainty in our estimates of global insect richness (2), and we agree that the greater the number of inferential steps lacking quantified measures of uncertainty, the less rigorous the final estimate. For example, Erwin’s contested approach [(3), cited by F&B] required no less than seven steps (4).

As F&B correctly summarize, our strategy for estimating global insect richness required only three inferential steps: i) estimation of the true richness of the hyperdiverse braconid wasp subfamily Microgastrinae in the Área de Conservación Guanacaste (ACG) in Costa Rica, including species present but undetected; ii) estimation of the true richness of all ACG insects (332,846), based on 1,633,855 barcoded specimens of 53,945 species from “core” Malaise traps, adjusted for undersampling by the ratio between the estimated true richness of Microgastrinae (2,394 [2,221; 2,604]) and the observed number of microgastrine species in the core Malaise samples (380 species); and iii) upscaling ACG insect richness to a global estimate, using multiple alternative ratios between published global estimates and expert ACG estimates of richness separately for trees, amphibians, mammals, and saturniid moths.

The first step yields a statistically rigorous, lower-bound estimate with 95% confidence intervals (5, 6). The second divides this estimate by a fixed count (zero variance) of Microgastrinae species in the core samples**—**a linear rescaling of a CI by a constant, not a new source of uncertainty. The resulting confidence interval is propagated to the third step to yield *conditional* confidence intervals on the global estimates of insect richness—conditional on point estimates of global and ACG richness for the four reference groups, whose own uncertainty is not further propagated. Each of these four alternative reference groups has its own level of uncertainty, which affects the final estimates by increasing the uncertainty inherited from the previous step but does not, on average, shift the center of the confidence interval. When this uncertainty was known, we included it—a range of ACG tree and amphibian species richness estimates and alternative estimates of global tree richness. The four reference groups thus provide a sensitivity analysis, not a statistical CI. In short, F&B’s concern that our estimate is “...determined not by a single estimator but by the combined action of several successive inferential operators...” is overstated: Uncertainty beyond the rigorous CI of the first step enters only through the third step.

In summary, because we were aware of the problem of propagated uncertainty in multistep estimation, we designed the study to rely on the absolute minimum number of inferential steps, capped by a sensitivity analysis across upscaling reference groups where quantitative uncertainty could not be estimated. The range of our estimates of global insect richness is wide, but even the lowest estimate—14 million species—is more than twice the previous consensus estimate. Moreover, our assumptions about collection methods, DNA barcode-based species delimitation, and biogeographical comparisons were—at every turn—far more likely to bias our estimates downward than upward.

## Data Availability

There are no data underlying this work.
